# Oxymatrine Attenuates Dopaminergic Neuronal Damage and Microglia-Mediated Neuroinflammation Through Cathepsin D-Dependent HMGB1/TLR4/NF-κB Pathway in Parkinson’s Disease

**DOI:** 10.3389/fphar.2020.00776

**Published:** 2020-05-26

**Authors:** Ping Gan, Lidong Ding, Guihua Hang, Qiaofang Xia, Zhimei Huang, Xing Ye, Xiaojuan Qian

**Affiliations:** Department of Pharmacy, Taizhou Second People’s Hospital, Taizhou, China

**Keywords:** Parkinson’s disease, oxymatrine, neuroinflammation, microglia, neuroprotection, cathepsin D

## Abstract

Oxymatrine (OMT), a natural quinoxaline alkaloid extracted from the root of *Sophora ﬂavescens*, presents amounts of pharmacological properties including immunomodulation, anti-inflammation, anti-oxidation, and anti-virus. Recent studies tend to focus on its effects on neuroinflammation and neuroprotection in Parkinson’s disease (PD) due to its profound anti-inﬂammatory effect. In this study, the neuroprotective and anti-neuroinflammatory effects of OMT were investigated in 1-methyl-4-phenyl-1, 2, 3, 6-tetrahydropyridine (MPTP)-stimulated mice and 1-methyl-4-phenylpyridinium (MPP^+^)-induced mice primary microglia. Additionally, mice primary neuron-microglia co-cultures and primary microglia infected with Cathepsin D (CathD)-overexpressed lentivirus were used to clarify whether the neuroprotective effect of OMT was through a CathD-dependent pathway. Results showed that OMT dose-dependently alleviated MPTP-induced motor deficits and conferred significant dopamine (DA) neuroprotection against MPTP/MPP^+^-induced neurotoxicity. In addition, OMT inhibited MPTP/MPP^+^-induced microglia activation and the pro-inflammatory cytokines release. Further, OMT down-regulated the expression of CathD, and inhibited the activation of the HMGB1/TLR4 signaling pathway as well as the nuclear translocation of NF-κB both *in vivo* and *in vitro*. It is worth noting that overexpression of CathD reversed OMT-targeted inhibition of HMGB1/TLR4/NF-κB signaling and OMT-produced neuroprotection in reconstituted neuron-microglia co-cultures. Our findings indicated that OMT conferred DA neuroprotection and attenuated microglial-mediated neuroinflammation through CathD-dependent inhibition of HMGB1/TLR4/NF-κB signaling pathway. Our study supports a potential role for OMT in ameliorating PD, and proposes that OMT may be useful in the treatment of PD.

## Introduction

Parkinson’s disease (PD), is pathologically characterized by progressive damage of dopamine (DA) neurons in the midbrain substantia nigra pars compacta (SNpc) (Ghosh et al., 2007). Although the pathogenesis of PD remains elusive, to date, an increasing number of evidence has shown that neuroinflammation mediated by microglia, astrocytes, macrophages, and mast cells, is a common factor in several neurodegenerative as well as neuropsychiatric diseases (Block and Hong, 2007; McKenzie et al., 2017; Novellino et al., 2020). Microglia, the most common immune cells in the central nervous system (CNS), has been considered to regulate the defense system after activated by an acute insult to the CNS (Kim and de Vellis, 2005). Once activated by either brain injury or neurotoxin exposure, microglia can produce and secrete various pro-inflammatory factors, including tumor necrosis factor-α (TNF-α), interleukin (IL)-1β, IL-6, and nitric oxide (NO). These inflammatory mediators not only contribute to the amplification of immune reactions, but also injure a great amount of DA neurons. Thus, inhibiting the microglial over-activation and the consequent neuroinflammation is likely to protect DA neurons from the chronic progression of PD.

The overactivation of high mobility group box 1 (HMGB1)/toll-like receptor 4 (TLR4) axis has been recognized as an important signaling pathway in inflammatory responses (Andersson et al., 2018). It has been reported that HMGB1 and TLR4 were highly expressing in the peripheral blood of PD patients compared with that in healthy controls (Yang et al., 2018), which demonstrated that an overaction of the HMGB1/TLR4 axis plays an essential role in PD development.

Cathepsin D (CathD) is now considered to participate in the long-term neuronal damage in PD (Yelamanchili et al., 2011). A previous study showed that CathD was significantly up-regulated and involved in trimethyltin-induced hippocampal neurodegeneration in rats, indicating the crucial role of CathD in this process (Ceccariglia et al., 2011). Therefore, down-regulation of CathD expression level is likely to promote the functional recovery in PD.

Oxymatrine (OMT), a natural quinoxaline alkaloid, is the primary biologically active component extracted from the root of *Sophora ﬂavescens*. OMT has been documented to bear a variety of pharmacological effects, including immunomodulation (Yao and Wang, 2014), anti-inflammation (Guzman et al., 2013), anti-oxidation (Jiang et al., 2015), anti-tumor (Fei et al., 2015), anti-virus, and hepatoprotection (Shi et al., 2013). Recently, experimental studies of OMT have focused on its effects on neuroinflammation and neuroprotection, mainly because of its anti-inflammatory property (Mao et al., 2012; Zhang et al., 2013). However, the exact role of OMT on neuroinflammation in PD and the underlying molecular mechanisms still remain to be clarified.

In this study, we investigated the neuroprotective effects of OMT against 1-methyl-4-phenyl-1, 2, 3, 6-tetrahydropyridine (MPTP) and 1-methyl-4-phenylpyridinium (MPP^+^)-induced DA neurotoxicity *in vivo* and *in vitro*, respectively. Additionally, the effects of OMT on the microglia activation and the subsequent neuroinflammatory reactions were also researched. Furthermore, the underlying mechanisms of CathD-dependent HMGB1/TLR4/NF-κB pathway were discovered using CathD-overexpressing mice primary microglia and neuron-microglia co-cultures.

## Materials and Methods

### Animals and Treatment

Male C57BL/6N mice (20–24 g, 8–10 weeks) purchased from Vital River (Beijing, China) were maintained under standard laboratory conditions on a 12 h light/dark cycle with food and water *ad libitum*.

In this study, 125 mice were randomly divided into: (1) Control group (n = 25): mice treated with sterilized physiological saline; (2) MPTP group (n = 25): mice continuously given an intraperitoneal injection (i.p.) of 25 mg/kg MPTP (M0896, Sigma-Aldrich, St. Louis, MO, USA) for 7 days (once a day) to establish the PD mice model followed by previous studies (Moriguchi et al., 2012; Patil et al., 2014; Lv et al., 2019); (3–5) OMT+MPTP groups (n = 25 in each experimental group): mice first given a daily injection with MPTP i.p. (25 mg/kg) 3 h later, followed by administration of OMT (dissolved in saline, PHL89748, Sigma-Aldrich) i.p. (5, 10, or 20 mg/kg) for 7 consecutive days. Finally, mice were sacrificed at day 8 (24 h after the last OMT administration) and the tissues were collected for further examinations. Five mice were randomly selected from each group for behavioral and histological examination. Five mice from each group were randomly selected for HPLC analysis. Seven mice from each group were randomly selected for ELISA, and eight mice were used for Western blot assay.

### Rotarod Test

The rotarod test was applied to detect the motor coordination. Briefly, mice were placed on an accelerating rotarod. Before the test, all mice were trained to stay from 0 revolutions per minute (rpm) a few minutes steadily to 10 rpm in 30 s interval, till they slid off the steps. Data was recorded from three repeated trials on 1 day, and the mean duration on the rod was finally calculated.

### Open Field Test

Mice were placed on the open field, any of which was separated in an independent area while the behavioral parameters were collected within 5 min. In order to get rid of the odor from each previous mouse, the instruments were cleaned with 75% ethyl alcohol before each next open field test. The distance of mice in central and peripheral activity areas was collected. Finally, the total distance of mice movement was calculated.

### Immunoﬂuorescence Assay

The brains tissues of mice were ﬁxed in 4% paraformaldehyde buffered with PBS at 4^°^C for 24 h, followed by changing into 30% sucrose solution for another 24 h. After embedding by optimum cutting temperature (O.C.T.) compound (Solarbio, Shanghai, China), the midbrain was cut into 16-μm-thick serial brain sections containing the SNpc for the subsequent immunoﬂuorescence staining. The slices were incubated with blocking solution (P0102, Beyotime, Beijing, China) including BSA and Triton X-100 for 1 h followed by the incubation overnight at 4^°^C with anti-tyrosine hydroxylase (TH, ab112, Abcam, Cambridge, MA, USA, 1:500) and anti-ionized calcium-binding adapter molecule 1 (Iba-1, ab178847, Abcam, 1:200) antibodies, respectively. The primary antibodies were diluted by QuickBlock™ Primary Antibody Dilution Buffer (P0262, Beyotime). Then the slices were incubated with secondary ﬂuorescent antibodies fluorescein isothiocyanate (FITC)-labeled Goat Anti-Rabbit IgG (green, A0562, Beyotime, 1:1,000) for 2 h. The secondary antibodies were diluted by QuickBlock™ Secondary Antibody Dilution Buffer (P0265, Beyotime). After that, fluorescence microscope (Carl Zeiss, Oberkochen, Germany) were applied to photograph.

### Measurement of the Number of TH^+^ Cells and the Fluorescence Intensity of Iba-1

The quantitative analysis of Immunoﬂuorescence assay was performed as previously described (Lanfranco et al., 2017; Takizawa et al., 2017). Briefly, for TH immunofluorescence staining, 10 areas were randomly selected from the brain tissue slides or mice primary microglia coverslips in each group. The total number of TH-positive DA neurons or cells from the randomly selected areas were all manually counted and divided by 10 to average. And the value was compared with other groups. For Iba-1 immunofluorescence staining, 10 fields were randomly selected from each brain tissue sections. Image Pro Plus 6.0 software (Mediacybernetics, MD, USA) was used to analyze the fluorescence intensity of Iba-1 immune positive cells.

### Western Blot Analysis

Cells and tissues were collected and total proteins were extracted by radioimmunoprecipitation assay (RIPA) Lysis buffer (Beyotime) containing 1% phenylmethylsulphonyl fluoride (PMSF, Beyotime) for 1 h on ice. After that, samples were centrifuged at 4^°^C. Then the protein concentration was determined by BCA assay kit (Beyotime). Nuclear protein was extracted by a nuclear and cytoplasmic protein extraction kit (P0028, Beyotime) following the manufacture’s protocol. Anti-TH antibody (ab112, Abcam, 1:1,000), anti-Iba-1 antibody (ab178847, Abcam, 1:500), anti-HMGB1 antibody (10829-1-AP, proteintech, Rosemont, IL, USA, 1:1,000), anti-TLR4 antibody (19811-1-AP, proteintech, 1:1,500), anti-CathD antibody (2284, Cell Signaling Technology, Danvers, MA, USA, 1:1,000), anti-p65 antibody (8242, Cell Signaling Technology, 1:1,000), anti-p-p65 antibody (3033, Cell Signaling Technology, 1:500), anti-Histone H3 antibody (proteintech, 1:1,500), and β-actin antibody (bsm-33139M, Bioss, Beijing, China, 1:2,000) were used as the primary antibodies in this study. All the primary antibodies were diluted by 1% BSA buffered with TBST. The membranes were blocked with 5% BSA buffered with TBST for 2 h at room temperature and incubated with one of the primary antibodies at 4^°^C overnight. The horseradish peroxidase (HRP)-conjugated goat anti-rabbit or anti-mouse IgG (Beyotime) was used as the secondary antibody, which as diluted by TBST. The enhanced chemiluminescence (ECL) was used for the specific protein bands visualization. To analyze western blot membranes, the Gel-Pro-Analyzer software (Version 4.0, Media Cybernetics, Rockville, MD, USA) were applied.

### High-Performance Liquid Chromatography (HPLC) Analysis

HPLC analysis was to detect the contents of DA and dihydroxy-phenyl acetic acid (DOPAC) in the striatum of mice. Briefly, the striatum were rapidly dissected and sonicated. After mixture with borate buffer, AccQ-Fluor reagent (WAT052880, Waters, Milford, USA) was added to 100 μl. Samples were then centrifuged for 30 min at 12,000 g, and the supernatants were finally filtered by 0.22 μm microfilters (Millex, Barcelona, Spain) before analyzing by HPLC. In this study, The Waters HPLC included a system controller (600E, Waters), a quaternary pump (2535, Waters), a fluorescence detector (2475 FLR Detector, Waters), and an autosampler (2707 Autosampler, Waters). Millenium software (Waters) was applied for collecting data, calculating peak area values, and controlling the system. The silica particle C8 Symmetry column (5 μm) with a 100-Å pore (Waters) was used for separation. The mobile phase included EDTA-2H_2_O (pH 2.65, 0.027 mM), NaH_2_PO_4_ (0.1 M), potassium chloride (2 mM), 18% methanol, and octyl sodium sulphate (1 mM). The flow rate of mobile phase was set at 1.0 ml/min. Fluorescence excitation was set at 252 nm, while the emission was determined at 397 nm.

### Measurement of Pro-Inflammatory Cytokines

The ventral midbrain tissues containing the SNpc, was first collected for homogenizing in PBS on ice. After centrifugation, the supernatant was used for detection. For cell experiment, cells were firstly centrifuged at 1,000 *g* for 20 min. Subsequently, the supernatant was used for detection. Levels of TNF-α, IL-1β, and IL-6 in tissues or cells were tested by ELISA kits (PAA133Mu01/KSA563Mu01/SEA079Mu, USCN Life Science, Wuhan, China) according to manufacturer’s protocols, respectively. The absorbance was read at 450 nm using a microplate reader (BIOTEK, Winooski, USA).

### Measurement of NO Levels

NO production in ventral midbrain or cells was analyzed by Total Nitric Oxide Assay Kit (Beyotime). After centrifugation, supernatant (50 μl) was mixed with Griess reagent I (50 μl), and then with Griess reagent II (50 μl) at room temperature for 10 min. The absorbance was set at 540 nm by a microplate reader (BIOTEK).

### Isolation of Mice Primary Microglia

Primary microglia was isolated from cerebral cortex tissues of newborn (1–2 days postnatal) C57BL/6N mice in this study. In brief, cerebral cortex tissues were collected and cultured in HBSS-Hank’s solution (Thermo Fisher, Waltham, USA). Then the meninges were totally removed from cerebral cortex. After chopping, tissues were transferred into 0.125% trypsin-EDTA (Thermo Fisher) at 37°C for 20 min. Subsequently, the cells were seeded in poly-L-lysine-coated flasks and cultured in DMEM/F12 (Gibco^@^, Grand Island, USA) with 10% FBS (Hyclone, Logan, USA) at 37^°^C containing 5% CO_2_. Culture medium was changed every 3–4 days. Fourteen days later, the microglial cells loosely adherent were recovered and isolated by shaking at 250 rpm at 37^°^C for 4 h. The isolated microglial cells were finally seeded into 6- or 24-well plates for mono-cultures. Three days later, either MPP^+^ (0.1 mM, Sigma-Aldrich, St. Louis, MO, USA) or OMT was added to primary microglia cultures. For MPP^+^ stimulation, mice primary microglia was pretreated with 0.1 mM MPP^+^ and incubated for 6 h (Koutsilieri et al., 1993; Zhou et al., 2016; Lv et al., 2019), followed by treatment with 0.2, 1, or 5 μM OMT for 24 h.

### Lentivirus Infection

The lentiviruses were purchased from Gene Pharma (Shanghai, China). CathD-OV primary microglia cells were infected with lentiviral particles overexpressing CathD and CathD-NC primary microglia cells were infected with negative control lentiviral particles. Uninfected primary microglia cells were the parental cells.

For lentivirus infection, primary microglia cells were seeded in six-well plates. The lentivirus diluted in DMEM/F12 medium (serum and antibiotic free) was added into cell culture to infect primary microglia cells. Twenty-four hours later, culture medium containing lentivirus was discarded, and cells were cultured in DMEM/F12 medium with 10% FBS.

### Real-Time PCR

Total RNA was extracted by RNAqueous^@^ Kit (ThermoFisher) and treated with RNase-free DNase I (Sigma-Aldrich). The concentration was tested by an ultraviolet spectrophotometer (ThermoFisher). Super M-MLV reverse transcriptase (PR6502, BioTeke, Beijing, China) was used to synthesize the complementary DNAs (cDNAs) from RNA templates. The 20 μl cDNAs were then amplified by 2×Power Taq PCR MasterMix (PR1702, BioTeke) and SYBR Green (SY1020, Solarbio, Beijing, China). Primers used in this study were as follows: CathD: 5′-CGCAGTGTTTCACAGTCGT-3′ (sense) and 5′-TGAGCCGTAGTGGATGTCAA-3′ (antisense); β-actin, 5′-CTGTGCCCATCTACGAGGGCTAT-3′ (sense) and 5′-TTTGATGTCACGCACGATTTCC-3′ (antisense). PCR reaction was performed on Exicycler™ 96 Thermal Block (Bioneer, Daejeon, Korea). The PCR amplification conditions were as follows: 94^°^C for 10 min, 40 cycles of denaturation at 94^°^C for 20 s, annealing at 60^°^C for 10 s, and extension at 72^°^C for 30 s, then 4^°^C for 5 min. Data was calculated by the 2^-ΔΔC^_T_ method.

### Isolation of Mice Primary DA Neuron

Primary DA neurons were isolated from the ventral mesencephalic tissues of C57BL/6N mice at gestation days 1–2. Briefly, mesencephalons were put in cold PBS and cut into small pieces. The tissues were transferred and incubated in 0.02% DNase I (Roche, Germany) and 0.1% Trypsin-EDTA (Thermo Fisher) at 37^°^C for 20 min. Then the digested tissues were incubated with DMEM medium (Gibco^@^). The digested cells were lightly filtered through 100 mesh filter, and single cells were resuspended in poly-L-lysine-coated flasks and cultured in DMEM medium supplied with 10% FBS (Hyclone A) at 37^°^C. Ten days later, either MPP^+^ or primary microglial cells were added to primary neuron cultures.

### Neuron-Microglia Reconstituted Cultures by Transwell

Neuron-microglia co-cultures were performed by Transwell assay. In brief, primary neuron cultures were first seeded into 24-well plates, while primary microglia cultures were seeded into transwell for 24 h. Then, the primary microglia cultures in transwell were infected by CathD-NC or CathD-OV lentiviral particles for another 24 h. Subsequently, the primary microglia cultures infected by CathD-NC or CathD-OV lentivirus were transferred to primary neuron cultures within fresh DMEM medium. Finally, the reconstituted neuron-microglia co-cultures were pretreated with MMP^+^ for 6 h, followed by treatment with OMT for 24 h. DA neuronal damage was evaluated by immunofluorescent staining and TH expression detection.

### Immunofluorescence Staining in Cells

The collected cells were fixed with 4% paraformaldehyde in PBS for 15 min at room temperature, permeabilized with 0.3% Triton X-100 for 30 min, and then blocked by 1% goat serum. After that, cells were treated with anti-TH (Abcam, 1:500) or anti-Iba-1 (Iba-1, Abcam, 1:200) antibody at 4^°^C overnight, followed by incubation with secondary antibody FITC-labeled goat anti-rabbit IgG (Beyotime, 1:1,000) at room temperature for 2 h. Fluorescence microscope (CX43, Olympus, Japan) was applied to observe the cell morphology of anti-TH-labeled DA neurons. Numbers of TH-positive neurons were recorded from three randomly selected areas of three parallel wells for each group.

### Statistical Analysis

Data in this study were expressed as the mean ± standard error of the mean (SEM) and analyzed using Graph Pad Prism software (Version 6.0, GraphPad software, San Diego, CA, USA). Each group is drawn from a normally distributed population. Comparison of two means was performed by Student’s *t* test, while multiple comparisons were analyzed by one-way analysis of variance (ANOVA) as well as Bonferroni’s *post-hoc* test. Statistical significance was set at *P* < 0.05.

## Results

### OMT Attenuated MPTP-Induced DA Neuronal Damage and Alleviated Motor Deficits in PD Mice Model

**Figure 1:**
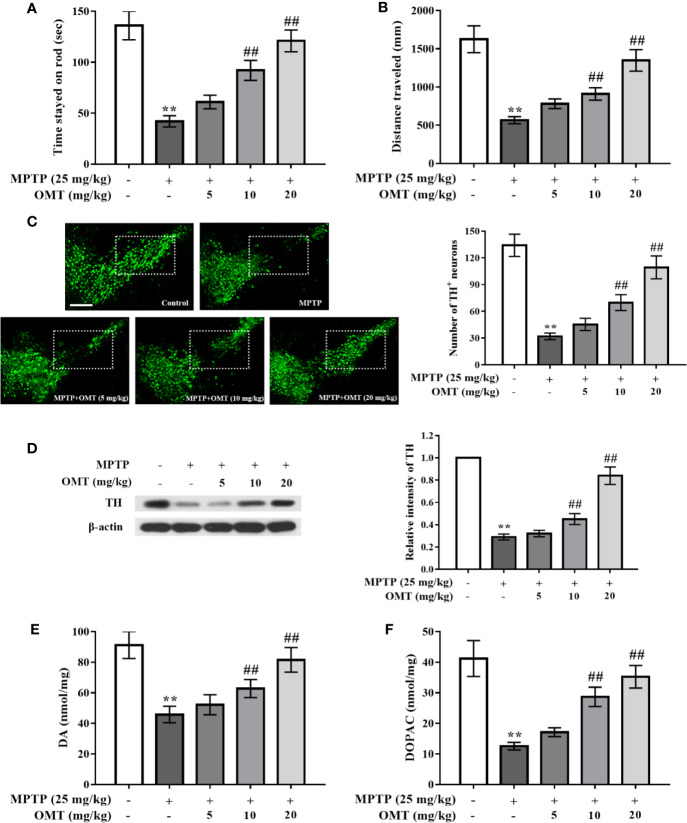
Oxymatrine (OMT) attenuated 1-methyl-4-phenyl-1, 2, 3, 6-tetrahydropyridine (MPTP)-induced dopamine (DA) neuronal damage and alleviated motor deficits in Parkinson’s disease (PD) mice model. Mice were first given a daily intraperitoneal injection (i.p.) of MPTP (25 mg/kg), followed by administration of OMT (i.p.) for 7 consecutive days. On day 8, rotarod test **(A)** and open-field test **(B)** were performed. **(C)** Tyrosine hydroxylase (TH)-immunopositive (green) neurons in substantia nigra pars compacta (SNpc) area were detected by immunofluorescence staining. Scale bar: 200 μm. **(D)** The expression of TH in midbrain tissues was determined by Western blot analysis. **(E**, **F)** Changes in DA system in midbrain tissues of MPTP-induced mice. Levels of DA **(E)** and its metabolite dihydroxy-phenyl aceticacid (DOPAC) **(F)** in striatal homogenates of respective groups were tested by HPLC analysis. The data are presented as mean ± SEM. ^**^p < 0.01 *vs.* Control group; ^##^p < 0.01 *vs.* MPTP group. ANOVA with Bonferroni’s *post-hoc* test.

The neuroprotection of OMT on MPTP-induced DA neuronal loss were studied in PD mice model. Firstly, the behavior changes of mice in respective groups were detected by rotarod and open-field tests (**Figures 1A, B**). It was found that compared with the control group, MPTP significantly induced the reduction of standing time during which mice stayed on the rod and the locomotor distance. The time mice lingered on the rotor was decreased from 135.63 ± 14.03 s in control group to 42.23 ± 5.60 s in MPTP-treated group. And the locomotor distance was also reduced from 1,625.69 ± 175.6 mm in control group to 567.78 ± 45.99 mm in MPTP-treated group. However, OMT (5, 10, 20 mg/kg) treatment dose-dependently attenuated MPTP-induced abnormal behavior, suggesting an improvement after OMT administration in motor coordination and total locomotor activity of PD mice (P < 0.01, **Figures 1A, B**). In addition, DA neuronal damage was tested by TH^+^ immunostaining *via* immunoﬂuorescence assay and TH protein expression through Western blot assay. As shown in **Figure 1C**, strong TH^+^ immunostaining in midbrain SNpc was clearly observed in control group, whereas the immunostaining of TH^+^ was obviously reduced in MPTP group. Administration of OMT (5, 10, 20 mg/kg) protected DA neurons from MPTP-induced neurotoxicity as shown by the enhanced TH^+^ immunostaining as well as the TH^+^ neuronal number in a dose-dependent manner (P < 0.01, **Figure 1C**). The further quantification of DA neurons also confirmed similar observations by Western blot assay. Data showed that the TH expression was markedly down-regulated by MPTP, which was up-regulated by OMT in a dose-dependent manner (P < 0.01, **Figure 1D**). Then the content of striatum DA and DOPAC was analyzed by HPLC assay. It was noticed that MPTP significantly induced the reduction of DA and DOPAC levels in striatum compared with control group. However, OMT increased both DA and DOPAC levels in a dose-dependent manner (P < 0.01, **Figures 1E, F**). These results indicated that OMT could improve motor deficits and protect DA neurons in MPTP-induced PD mice.

### OMT Attenuated Microglia Activation and Suppressed Pro-Inflammatory Cytokines Production in MPTP-Induced PD Mice Model

**Figure 2:**
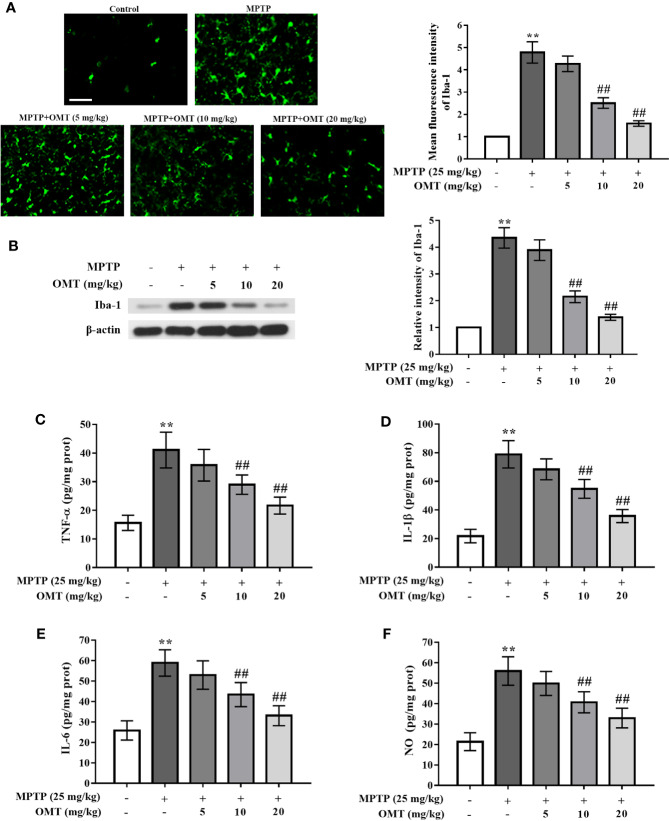
OMT attenuated microglia activation and suppressed pro-inflammatory cytokines production in MPTP-induced PD mice model. **(A)** Immunofluorescence staining for Iba-1 positive microglia in SNpc area after administration with OMT in MPTP-induced PD mice. Scale bar: 100 μm. **(B)** The expression of Iba-1 in midbrain was determined by Western blot analysis. **(C–F)** Levels of pro-inflammatory cytokines, such as TNF-α **(C)**, IL-1β **(D)**, IL-6 **(E)**, and NO **(F)** in midbrain were tested by ELISA **(C–E)** and Griess **(F)** regent, respectively. The data are presented as mean ± SEM. ^**^p < 0.01 *vs.* Control group; ^##^p < 0.01 *vs.* MPTP group. ANOVA with Bonferroni’s *post-hoc* test.

The microglia activation induced by MPTP was detected by Iba-1^+^ (a marker of microglia) immunostaining *via* immunoﬂuorescence assay and Iba-1 protein expression through Western blot assay. Data showed that MPTP markedly induced microglia activation compared with control group. However, OMT (5, 10, 20 mg/kg) suppressed MPTP-induced microglia activation in a dose-dependent manner (P < 0.01, **Figure 2A**). In addition, western blot analysis also revealed that the up-regulated Iba-1 expression induced by MPTP was inhibited by OMT (5, 10, 20 mg/kg) in a dose-dependent manner (P < 0.01, **Figure 2B**).

To further assess the inhibitory effect of OMT on the production of microglial pro-inflammatory cytokines, the concentration levels of TNF-α, IL-1β, IL-6 and NO in the midbrain tissues of MPTP-induced PD mice were tested by ELISA analysis. In contrast to MPTP-treated mice, OMT (5, 10, 20 mg/kg) administration remarkably decreased the production of these pro-inflammatory cytokines in a dose-dependent manner (P < 0.01, **Figures 2C–F**). These data demonstrated that OMT could attenuate microglia activation and the consequent neuroinflamamtion in MPTP-induced PD mice.

### OMT Down-Regulated CathD Expression and Inhibited HMGB1/TLR4/NF-κB Signaling Pathway in MPTP-Induced PD Mice Model

**Figure 3:**
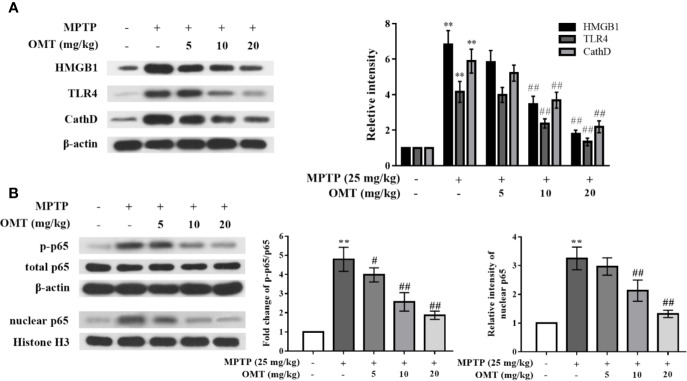
OMT down-regulated Cathepsin D (CathD) expression and inhibited HMGB1/TLR4/NF-κB signaling pathway in MPTP-induced PD mice model. **(A**, **B)** The expression levels of CathD, HMGB1, TLR4 (A), p-p65, and total p65 whole cell expression as well as p65 nuclear expression **(B)** in midbrain of MPTP-induced PD mice were tested by Western blot analysis. The data are presented as mean ± SEM. ^**^p < 0.01 *vs.* Control group; ^##^p < 0.01 *vs.* MPTP group ^#^p < 0.05 vs. MPP+-treated cells. ANOVA with Bonferroni’s *post-hoc* test.

To confirm whether OMT-targeted inhibition of microglia activation was mediated through the HMGB1/TLR4/NF-κB signaling pathway, firstly, the expressional levels of HMGB1, TLR4, and CathD were evaluated by Western blot analysis in midbrains of MPTP-induced PD mice. As shown in **Figure 3A**, expression levels of HMGB1, TLR4, and CathD were remarkably up-regulated after MPTP injection. In addition, phosphorylation level of p65 and nuclear p65 expression were also obviously up-regulated in MPTP-induced mice (**Figure 3B**). However, OMT (5, 10, 20 mg/kg) not only down-regulated the expression of HMGB1, TLR4, and CathD, but also inhibited p65 phosphorylation and nuclear translocation in a dose-dependent manner (P < 0.01, **Figures 3A, B**).

### OMT Suppressed Pro-Inflammatory Cytokines Production and Inhibited HMGB1/TLR4/NF-κB Activation in MPP^+^-Induced Mice Primary Microglia

**Figure 4:**
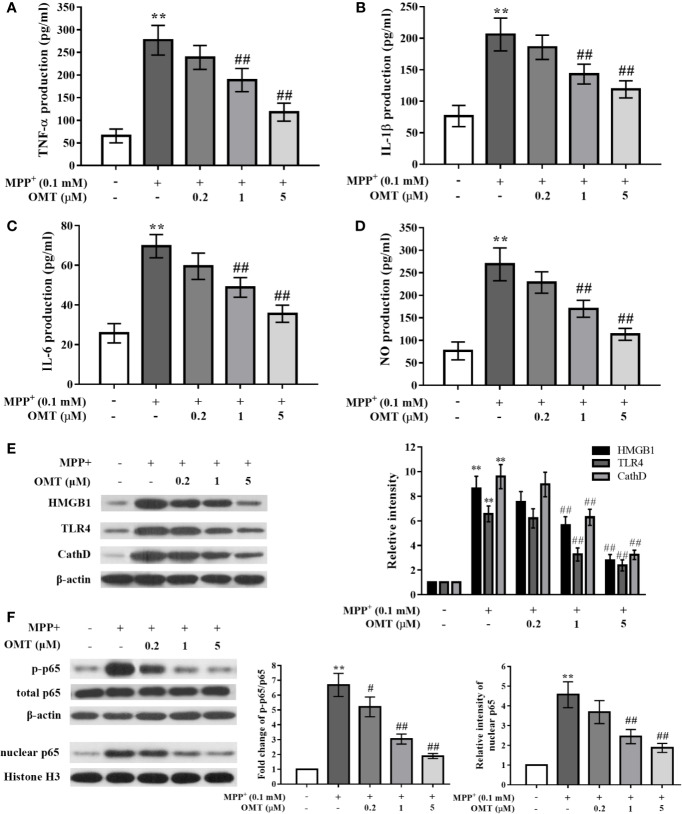
OMT suppressed proinflammatory cytokines production and inhibited HMGB1/TLR4/NF-κB activation in MPP^+^-induced mice primary microglia. Mice primary microglia was pretreated with 0.1 mM MPP^+^ and incubated for 6 h, followed by treatment with 0.2, 1, or 5 μM OMT for 24 h. **(A–D)** Levels of pro-inflammatory cytokines, such as TNF-α **(A)**, IL-1β **(B)**, IL-6 **(C)**, and NO **(D)** in MPP^+^-induced primary microglia were tested by ELISA analysis. **(E**, **F)** The expression levels of CathD, HMGB1, TLR4 **(E)**, p-p65, and total p65 whole cell expression as well as p65 nuclear expression **(F)** in MPP^+^-induced primary microglia were tested by Western blot analysis. The data are presented as mean ± SEM. ^**^p < 0.01 *vs.* Control cells; ^##^p < 0.01 *vs.* MPP^+^-treated cells. ^#^p < 0.05 vs. MPP+-treated cells. ANOVA with Bonferroni’s *post-hoc* test.

To further explore the effects of OMT on neuroinflamamtion and the potential underlying mechanism, the anti-inflammatory ability of OMT was confirmed in MPP^+^-induced mice primary microglia *in vitro*. Data revealed that MPP^+^ significantly enhanced the production of pro-inflammatory factors including TNF-α, IL-1β, IL-6, and NO compared with control primary microglia by ELISA assay, whereas OMT (0.2, 1, 5 μM) suppressed the secretion of these cytokines a dose-dependent manner (P < 0.01, **Figures 4A–D**). Moreover, in the detection of signaling pathway by Western blot analysis, we also found that the HMGB1/TLR4/NF-κB pathway was activated by MPP^+^ induction, together with remarkably up-regulation of CathD. However, OMT (0.2, 1, 5 μM) inhibited the activation of HMGB1/TLR4/NF-κB pathway and down-regulated the expression of CathD in a dose-dependent manner (P < 0.01, **Figures 4E, F**). Additionally, the effects of OMT alone on the survival of DA neurons and the activation of microglia were detected by immunofluorescence analysis. In **Supplementary Figure 1**, data showed that different doses of OMT alone neither influenced the number of DA neurons, nor altered the activation of microglia. Taken together, these data indicated that OMT suppressed microglia activation and pro-inflammatory cytokines production induced by MPP^+^ probably through down-regulation of CathD and inhibition of HMGB1/TLR4/NF-κB signaling pathway.

### Overexpression of CathD Reversed OMT-Targeted Inhibition of HMGB1/TLR4/NF-κB Signaling Pathway in MPP^+^-Induced Primary Microglia

**Figure 5:**
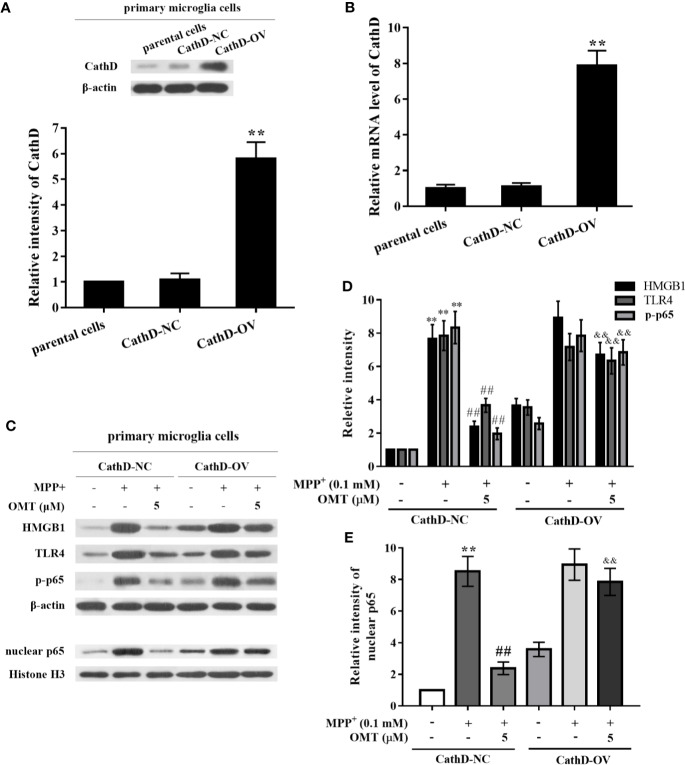
OMT inhibited HMGB1/TLR4/NF-κB signaling pathway through down-regulation of CathD in MPP^+^-induced primary microglia. Mice primary microglia cells were infected with negative control lentiviral particles (CathD-NC) or lentiviral particles overexpressing CathD (CathD-OV). The mRNA and protein levels of CathD were determined by Western blot **(A)** and real-time RT-PCR **(B)** analysis. Twenty-four hours later, cells were pretreated with 0.1 mM MPP^+^ and incubated for 6 h, followed by treatment with 5 μM OMT for another 24 h. **(C–E)** The activation of HMGB1/TLR4/NF-κB signaling pathway after overexpression of CathD was tested by Western blot analysis in MPP^+^-induced primary microglia cells. The data are presented as mean ± SEM. ^**^p < 0.01 *vs.* CathD-NC cells; ^##^p < 0.01 *vs.* MPP^+^-treated CathD-NC cells; ^&&^p < 0.01 *vs.* MPP^+^- and OMT-treated CathD-NC cells. Student’s *t* test.

In order to investigate the role of CathD on OMT-targeted inhibition of HMGB1/TLR4/NF-κB signaling pathway, mice primary microglia infected with CathD-overexpressed lentivirus were further conducted in this study. Firstly, mice primary microglia cells were infected with CathD-overexpressed lentivirus (CathD-OV) or empty lentivirus (CathD-NC). The CathD-overexpressing efficiency was tested by Western blot and real time-PCR analysis. Data showed that CathD-OV significantly increased protein expression of CathD in primary microglia, while CathD-NC did not. Although there was no significant difference between parental cells and CathD-NC-infected primary microglia, the mRNA level of CathD-OV-infected primary microglia was almost eighth times compared to CathD-NC-infected cells (P < 0.01, **Figures 5A, B**). Next, we found that OMT-mediated down-regulation of HMGB1, TLR4, and CathD, and the inhibition of p65 phosphorylation and nuclear expression were remarkably reversed in MPP^+^-induced CathD-OV primary microglia compared with CathD-NC primary microglia (P < 0.01, **Figures 5C–E**). Thus, these data suggested that CathD participated in OMT-targeted inhibition of HMGB1/TLR4/NF-κB signaling pathway in MPP^+^-induced primary microglia.

### Overexpression of CathD Reversed OMT-Produced Neuroprotection in Reconstituted Neuron-Microglia Co-Cultures

**Figure 6:**
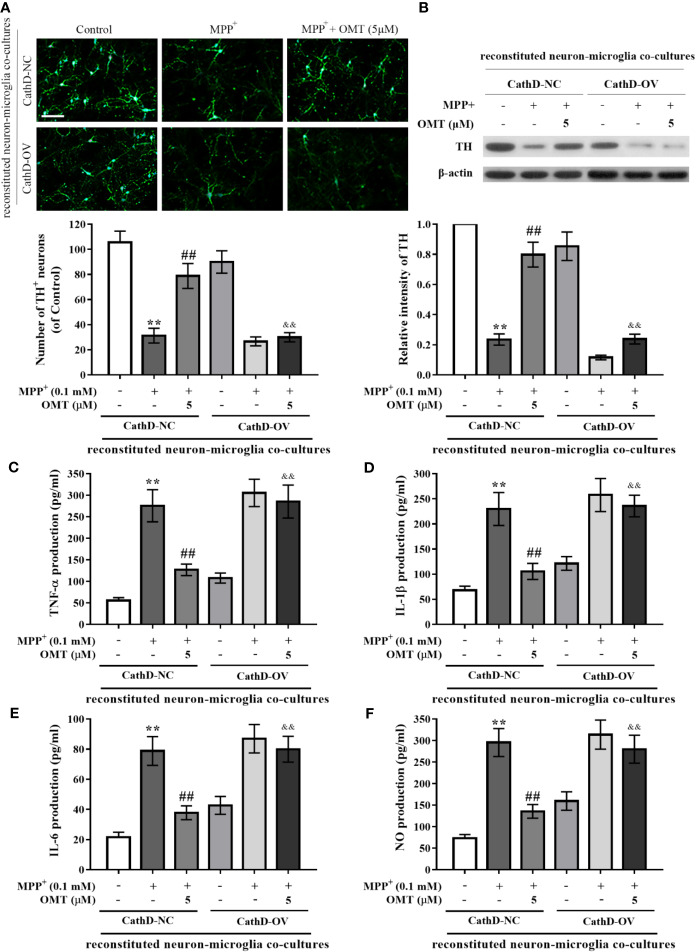
Overexpression of CathD reversed OMT-produced neuroprotection in reconstituted neuron-microglia co-cultures. Mice primary microglia after overexpression of CathD was transferred to neuron-enriched cultures. After that the reconstituted neuron-microglia co-cultures were pretreated with MMP^+^ for 6 h, followed by treatment with 5 μM OMT for 24 h. **(A)** DA neurons quantification was determined by TH-immunofluorescence staining. Scale bar: 100 μm. **(B)** The expression of TH in reconstituted neuron-microglia co-cultures was determined by Western blot analysis. **(C–F)** Levels of pro-inflammatory cytokines, such as TNF-α **(C)**, IL-1β **(D)**, IL-6 **(E)**, and NO **(F)** in reconstituted neuron-microglia co-cultures were tested by ELISA **(C-E)** and Griess **(F)** analysis. The data are presented as mean ± SEM. ^**^p < 0.01 *vs.* CathD-NC reconstituted co-cultures; ^##^p < 0.01 *vs.* MPP^+^-treated CathD-NC reconstituted co-cultures; ^&&^p < 0.01 *vs.* MPP^+^- and OMT-treated CathD-NC reconstituted co-cultures. Student’s *t* test.

To further investigate the neuroprotective effect of OMT, mice primary neuron-microglia co-cultures were applied. Mice primary microglia cells after infected with CathD-OV or CathD-NC lentivirus were transferred to primary neuron cultures. Subsequently, reconstituted neuron-microglia co-cultures were firstly stimulated by MPP^+^, and then treated with 5 μM OMT. DA neurons immunostaining and TH protein expression measurement were detected by immunoﬂuorescence and Western blot assays, respectively. Data indicated no significant difference between CathD-NC and CathD-OV reconstituted neuron-microglia co-cultures in Control groups. Accordingly, MPP^+^ significantly reduced DA neuronal number and TH protein expression in both CathD-NC and CathD-OV reconstituted neuron-microglia co-cultures in MPP^+^ groups. In addition, MPP^+^-induced reduction of TH^+^ DA neuronal number as well as down-regulation of TH expression was remarkably ameliorated by OMT treatment. However, overexpression of CathD reversed OMT-protected DA neurons against MPP^+^-induced neurotoxicity in CathD-OV reconstituted neuron-microglia co-cultures compared with CathD-NC reconstituted neuron-microglia co-cultures (P < 0.01, **Figures 6A, B**). Moreover, overexpression of CathD also blocked the inhibitory effect of OMT on MPP^+^-induced secretion of pro-inflammatory cytokines TNF-α, IL-1β, IL-6, and NO in CathD-OV reconstituted neuron-microglia co-cultures compared with CathD-NC reconstituted neuron-microglia co-cultures by ELISA assay (P < 0.01, **Figures 6C–F**). Collectively, these results indicated that OMT inhibited HMGB1/TLR4/NF-κB signaling pathway through down-regulation of CathD to exert DA neuroprotection in MPP^+^-induced primary microglia.

## Discussion

The present study investigated the neuroprotective effects of OMT on MPTP-/MPP^+^-induced DA neurotoxicity both *in vivo* and *in vitro*, respectively and the underlying mechanisms related with the neuroinflammation in PD. Results demonstrated that OMT dose-dependently alleviated MPTP-induced motor deficits and conferred significant DA neuroprotection against MPTP/MPP^+^-induced neurotoxicity. In addition, OMT inhibited MPTP/MPP^+^-induced microglia activation and the pro-inflammatory cytokines release. Further, OMT down-regulated the expression of CathD, inhibited the activation of the HMGB1/TLR4 signaling pathway, and suppressed the nuclear translocation of NF-κB both *in vivo* and *in vitro*.

It has been well established that neuroinflammation and DA neuronal damage in the SNpc neurons are typical characteristics of PD (Glass et al., 2010). As the resident immune cells in brains, microglia play an essential role in the neuroinflammation. Positron emission tomography (PET) studies have demonstrated that the activation of microglia is widespread in the brains of PD patients (Gerhard et al., 2006; Bartels et al., 2010; Fan et al., 2015; Koshimori et al., 2015). Additionally, the activation of microglia is also basically typical in the SNpc and striatum in different kinds of PD animal models (Tansey et al., 2007; Benner et al., 2008). Ouchi et al. reported that the activation of microglia in the midbrain tissues has been considered to be positively correlated with the injury of dopaminergic neurons and the motor deficit in PD (Ouchi et al., 2009). The activated microglia can release various kinds of pro-inflammatory cytokines, such as TNF-α, IL-1β, and IL-6, which may contribute to hastening neuronal dysfunction and degeneration of DA neurons, thereby accelerating PD progression. It is interesting to note that the anti-inflammatory effect of OMT has been focused in recent studies (Mao et al., 2012; Ding et al., 2016). Further, OMT has also found to prevent cerebral ischemic injury induced by middle cerebral artery occlusion (MCAO) *in vivo* and to produce neuroprotection against N-methyl-D-aspartate (NMDA)-induced neurotoxicity *in vitro* (Cui et al., 2011; Zhang et al., 2013). In our study, data showed that OMT improved motor deficits and protected DA neurons from MPTP-induced neurotoxicity in PD mice. Moreover, the activation of microglia and inflammatory responses were both inhibited by OMT in the midbrains of MPTP-induced PD mice. OMT also dose-dependently suppressed the production of pro-inflammatory cytokines in MPP^+^-induced mice primary microglia *in vitro*. Since MPP^+^ is the metabolite of MPTP (Markey et al., 1984), data suggested that microglia is likely to be the targeted cells of OMT in PD treatment. Together, our results indicated that OMT ameliorated PD symptoms primarily through inhibition of neuroinflammation.

HMGB1 has been found to be closely related to the inflammatory diseases (Lu et al., 2012). It can be actively secreted by inflammatory cells and released by necrotic cells (Angelopoulou et al., 2018). After that HMGB1 can bind to various transmembrane receptors including TLR2, TLR4, and TLR9, and increase DNA-binding capacity of several transcription factors, such as p53 as well as NF-κB (Martinotti et al., 2015), ultimately leading to inflammatory responses. Nowadays, HMGB1/TLR4/NF-κB axis has been demonstrated as the key pro-neuroinflammatory signaling pathway in CNS (Chen et al., 2017; Takizawa et al., 2017). Fujita et al. found that intracerebroventricular injection of HMGB1 could enhance the secretion of pro-inflammatory factors in brains of mice (Fujita et al., 2016). Clinical study also reported that both expression of HMGB1 and TLR4 was remarkably up-regulated in PD patients compared with healthy controls, together with that the activation of NF-κB pathway and TNF-α level were positively correlated with high expression of the HMGB1/TLR4 axis (Yang et al., 2018). Mechanically, once astrocytes and microglia are activated, the released HMGB1 will further facilitates the activation of microglia to form a vicious circle *via* HMGB1/TLR4/NF-κB axis, thereby accelerating the neuroinflammatory responses in brains (Gao et al., 2011). Therefore, inhibition the activation of HMGB1/TLR4/NF-κB signaling pathway may be beneficial for PD treatment. Mariucci et al. found that the absence of TLR4 modified MPTP-induced DA neuronal loss in TLR^−/−^ mice compared with wild-type mice (Mariucci et al., 2018). In addition, studies have demonstrated the inhibitory effect of OMT on HMGB1/TLR4 or TLR4/NF-κB axis in other tissues or cells (Mao et al., 2012; Ding et al., 2016; Zhao et al., 2016). In line with these results, this study revealed that OMT down-regulated the expression levels of HMGB1, TLR4 and CathD in a dose-dependent manner. Furthermore, the activation of downstream NF-κB signaling was also inhibited by OMT treatment both *in vivo* and *in vitro*. These data indicated that OMT inhibited neuroinflammation and protected DA neurons *via* suppression of HMGB1/TLR4/NF-κB signaling pathway in PD mice model.

Additionally, the increased CathD expression may be relevant to HMGB1/TLR4 axis-regulated clearance of axon debris in hypertrophic microglia of cortical spreading depression (Takizawa et al., 2017). It has been well-demonstrated that CathD is closely involved in neuroinflammation and several neurodegenerative diseases (Roberg and Ollinger, 1998; Roberg et al., 1999; Yelamanchili et al., 2011; Amritraj et al., 2013). Liu et al. reported that the increased expression of CathD was observed earlier than the production of pro-inflammatory cytokines in oxygen-glucose deprivation/reperfusion-induced astrocytes (Liu et al., 2016). In addition, CathD was also found to be up-regulated in the activated microglia (Takenouchi et al., 2011; Yelamanchili et al., 2011), the increased level of which can cause neuronal death (Amritraj et al., 2013). Our previous study reported that CathD was significantly up-regulated in MPTP-induced PD mice *in vivo* and in LPS-induced mice primary microglia *in vitro*, and that knockdown of CathD inhibited neuroinflammation and protected DA neurons from microglia-mediated neurotoxicity *via* inhibiting the activation of NF-κB signaling pathway (Gan et al., 2018). Therefore, CathD not only participates in the neuroinflammatory responses, but also trigger neurodegeneration and possibly development of neurodegenerative disorders including PD. In the present study, we found that down-regulation of CathD closely involved in OMT-exerted neuroprotection, as evidenced by the following findings. First, OMT down-regulated CathD expression both *in vivo* and *in vitro*. Second, OMT-targeted inhibition of HMGB1/TLR4/NF-κB signaling pathway in MPP^+^-induced mice primary microglia was reversed by overexpression of CathD. Third, OMT exerted little DA neuroprotection and barely inhibit the production of microglia-mediated pro-inflammatory cytokines in reconstituted neuron-microglia co-cultures infected with CathD-overexpressed lentivirus. Together, OMT inhibited microglia-mediated neuroinflammation and afforded DA neuroprotection through CathD-dependent inhibition of HMGB1/TLR4/NF-κB pathway.

In conclusion, our findings demonstrate that OMT could effectively attenuate MPTP-induced PD by conferring DA neuroprotection and inhibiting microglial-mediated neuroinflammation through CathD-dependent inhibition of HMGB1/TLR4/NF-κB signaling pathway. These results provide experimental evidence indicating that OMT might be useful in the treatment of PD.

## Data Availability Statement

The datasets generated for this study are available on request to the corresponding author.

## Ethics Statement

The animal study was reviewed and approved by the Animal Care and Use Committee of Laboratory Animals of Chinese Academy of Science and Taizhou Second People’s Hospital.

## Author Contributions

PG and LD are joint first authors of the study. PG and XQ designed the experiments. PG, LD, GH, QX, and ZH performed the experiments. PG, LD, and GH performed the data analysis. QX, ZH, and XY contributed to the preparation of reagents and materials. PG and XQ wrote the manuscript. All authors read and approved the final manuscript.

## Funding

This study was supported by the grant from the Introduction Program of High-level Innovative and Entrepreneurial Talents of Jiangsu Province (No.: [2016] No.32).

## Conflict of Interest

The authors declare that the research was conducted in the absence of any commercial or financial relationships that could be construed as a potential conflict of interest.

## Supplementary Material

Supplementary Figure 1 The effects of OMT alone on the survival of DA neurons and the activation of microglia. **(A)** Mice primary DA neurons were treated with 0.2, 1, or 5 μM OMT for 24 h. DA neurons quantification was determined by TH-immunofluorescence staining. Scale bar: 100 μm. **(B)** Immunofluorescence staining for Iba-1 positive microglia in mice primary microglia cells treated with 0.2, 1, or 5 μM OMT for 24 h. Scale bar: 100 μm. The data are presented as mean ± SEM.
