# Supplementary figures and images for: Oxymatrine Attenuates Dopaminergic Neuronal Damage and Microglia-Mediated Neuroinflammation Through Cathepsin D-Dependent HMGB1/TLR4/NF-κB Pathway in Parkinson’s Disease
Source: Front Pharmacol. 2020 May 26;11:776. doi: 10.3389/fphar.2020.00776 (PMC7264119; doi:10.3389/fphar.2020.00776)

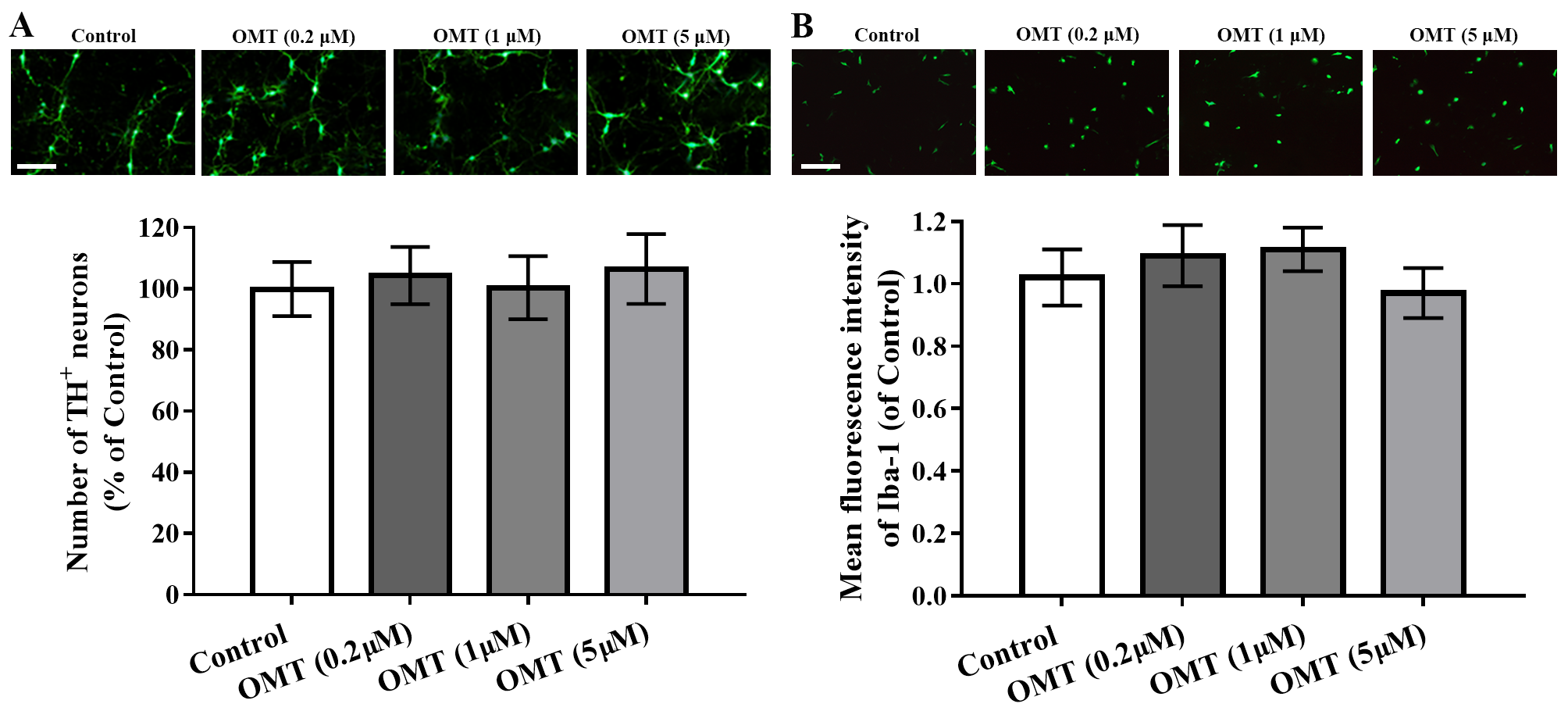

Supplement: Supplementary Figure 1 — The effects of OMT alone on the survival of DA neurons and the activation of microglia. (A) Mice primary DA neurons were treated with 0.2, 1, or 5 μM OMT for 24 h. DA neurons quantification was determined by TH-immunofluorescence staining. Scale bar: 100 μm. (B) Immunofluorescence staining for Iba-1 positive microglia in mice primary microglia cells treated with 0.2, 1, or 5 μM OMT for 24 h. Scale bar: 100 μm. The data are presented as mean ± SEM. [file Image_1.tif]
